# Maternal Paracetamol Intake During Pregnancy—Impacts on Offspring Reproductive Development

**DOI:** 10.3389/ftox.2022.884704

**Published:** 2022-04-14

**Authors:** Rieko Tadokoro-Cuccaro, Benjamin G. Fisher, Ajay Thankamony, Ken K. Ong, Ieuan A. Hughes

**Affiliations:** ^1^ Department of Paediatrics, University of Cambridge, Cambridge, United Kingdom; ^2^ Wellcome/Cancer Research UK Gurdon Institute, University of Cambridge, Cambridge, United Kingdom; ^3^ Cambridge University Hospitals NHS Foundation Trust, Cambridge, United Kingdom; ^4^ Wellcome-MRC Institute of Metabolic Science, University of Cambridge, Cambridge, United Kingdom; ^5^ MRC Epidemiology Unit, University of Cambridge, Cambridge, United Kingdom

**Keywords:** paracetamol, acetaminophen, anogenital distance, fetal exposure, reproductive development, endocrine disruption, toxicology

## Abstract

Paracetamol (acetaminophen) is the preferred antipyretic/analgesic for pregnant women as it is believed there are no adverse fetal effects at the recommended dose. However, emerging evidence suggests that intrauterine paracetamol exposure may be associated with certain urogenital/reproductive disorders in the offspring. In this mini-review, we describe human fetal sex development and possible pharmacological mechanisms by which paracetamol may disrupt this process, including reduced testicular production of testosterone and/or insulin-like peptide 3. We then review the available epidemiological literature on associations between maternal paracetamol exposure and offspring sexual development. Three epidemiological studies have reported associations between maternal paracetamol intake and increased risk of cryptorchidism, although five others have not. None have found associations with hypospadias or penile length. Two out of three studies have reported a shorter anogenital distance (a marker of androgen action during the masculinisation programming window, ∼8–14 weeks of gestation) in male infants antenatally exposed to paracetamol. One study has described a dose-dependent relationship between maternal paracetamol consumption and earlier female (but not male) attainment of puberty. Such epidemiological analyses are complicated by various factors, including method of paracetamol exposure assessment (usually retrospective self-report), variation in diagnostic accuracy, selection bias, confounding by clinical indication, and demographic/genetic differences between geographically separated populations. There is an urgent need for stronger evidence in this area, from both relevant experimental studies and large, carefully-designed prospective studies. In the meantime, a precautionary attitude to gestational paracetamol usage should be considered as the evidence for clinically significant reproductive effects in humans is limited.

## Introduction

Paracetamol (also known as acetaminophen or *N*-acetyl-*p*-aminophenol, APAP) is one of the most widely used antipyretics and analgesics in the world. It is available without prescription in most European countries and the United States. It is believed to have no significant adverse effects on the fetus at the recommended dose and duration and is the first-line treatment for these purposes over other anti-inflammatory medications in pregnant women as well as in children. Paracetamol is used by 50–70% of pregnant women in the Western world, with 15–54% taking it during the first trimester (Werler et al., 2005; Rebordosa et al., 2008; Kristensen et al., 2011). European and American guidelines recommend using the lowest dose only for as long as needed in pregnancy and consulting a healthcare professional if the medication is required more often (European Medicines Agency 2019; U.S. Food and Drug Administration 2015).

There is a concern that the prevalence of certain neurodevelopmental, urogenital, and reproductive disorders in children and adults has risen worldwide in the last few decades (Bauer et al., 2021). Some experimental and epidemiological research studies have suggested that paracetamol, which readily crosses the placenta (Rayburn et al., 1986), may adversely affect fetal development, which could increase the risk of these disorders. As a result, an international group of scientists, clinicians and epidemiologists has recently published a consensus statement that, paracetamol should be used more cautiously during pregnancy (Bauer et al., 2021). The statement was widely publicised in the media (Minet 2021; Gibbons 2021), and some authors have disagreed with the conclusions reached (Alwan et al., 2021; Damkier et al., 2021).

Studies of fetal exposure to paracetamol have focused on many areas: premature birth (Sujan et al., 2019), low birth weight (Arneja et al., 2020), learning and behavioural problems in childhood, including autism spectrum disorder (Brandlistuen et al., 2013; Avella-Garcia et al., 2016), genital anomalies (Jensen et al., 2010; Kristensen et al., 2011), other birth defects (Feldkamp et al., 2010; Marsh et al., 2014), and altered pubertal development (Ernst et al., 2019). The focus of this review is on human fetal and postnatal reproductive development in relation to maternal exposure to paracetamol.

## Sex Development in the Human Fetus

Sex development in humans starts in the early stages of gestation. If an embryo is a genetically male (46, XY), the presence of the *SRY* gene on the Y chromosome triggers the bipotential gonads to differentiate into testes, starting around week six post-fertilisation (Titi-Lartey and Khan, 2022). The Leydig cells of the testes then produce androgens which stimulate the development of the male internal genitalia (epididymis, vas deferens, and seminal vesicles) from the mesonephric ducts, and the male external genitalia (penis and scrotum) from the bipotential urethral folds, genital tubercle, and genital swellings; anti-mullerian hormone (AMH) from the Sertoli cells of the testes induces regression of the paramesonephric ducts (Titi-Lartey and Khan, 2022). Inadequate androgen action during weeks 8–12 can lead to incomplete fusion of the urethral folds resulting in hypospadias (Titi-Lartey and Khan, 2022). The testes descend from the posterior abdominal wall to the scrotum in two phases: a transabdominal phase during weeks 8–15, which requires the secretion of insulin-like peptide 3 (INSL3) from the Leydig cells; and an inguino-scrotal phase during weeks 25–35, in which testosterone plays an important role (Elamo et al., 2022). Failure of this process leads to cryptorchidism (undescended testis) (Hughes and Acerini 2008).

In females (46, XX), absence of gonadal *SRY* expression results in ovarian differentiation, orchestrated by ovary-specific transcription factors. A lack of AMH allows the paramesonephric ducts to persist and fuse with each other to form the female internal genitalia (oviducts, uterus, cervix, and upper vagina); a lack of androgens results in regression of the mesonephric ducts, and differentiation of the urethral folds, genital swellings, and genital tubercle into the labia minora, labia majora, and clitoris, respectively (Titi-Lartey and Khan, 2022).

The relatively low incidence of hypospadias and cryptorchidism in male newborns has led authors to explore other biomarkers of intrauterine reproductive development. One such biomarker of fetal androgen action is anogenital distance (AGD), which is easily measured, sexually dimorphic (longer in males than females from 11 to 13 weeks of gestation in humans), and responsive experimentally to anti-androgen exposures during gestational days 15.5–19.5 in rats, equivalent to approximately 8–14 weeks of gestation in humans—the so-called masculinisation programming window (MPW) (Welsh et al., 2008). In epidemiological studies, shortened AGD has been associated with cryptorchidism, hypospadias, reduced male typical behaviour at 3–4 years of age, and various adult male outcomes, including poor semen quality, infertility, low serum testosterone levels, decreased testicular volume, ineffective varicocele repair, and prostate cancer (Dean and Sharpe 2013; Thankamony et al., 2014; Pasterski et al., 2015). Penile length has also been shown to reflect androgen action during the MPW in rodents (Welsh et al., 2008), and short penile length has been associated with AGD at birth, hypospadias, and cryptorchidism in humans (Thankamony et al., 2009; 2014); however, unlike AGD, it is not associated with adult male outcomes (testicular volume, semen parameters, and serum testosterone levels) (Eisenberg et al., 2011, 2012; Khan et al., 2012).

## Pharmacological Effects of Paracetamol

Paracetamol has antipyretic and analgesic properties but lacks anti-inflammatory effects in peripheral sites, as its main site of pharmacological action is within the central nervous system. Paracetamol inhibits cyclooxygenase (COX)-2-dependent production of prostaglandins and can easily cross the blood brain barrier (Kumpulainen et al., 2007) and placenta, with equivalent serum concentrations in mother and fetus following a therapeutic dose (Naga Rani and Narayanan, 1989). Paracetamol’s analgesic properties are thought to additionally be mediated through the cannabinoid and vanilloid signalling pathways. The metabolite of paracetamol, *N*-arachidinoyl-phenolamine (AM404), acts as an agonist of cannabinoid receptors type 1 and 2, and an inhibitor of the anandamide membrane transporter, which leads to increased levels of endogenous cannabinoids. AM404 is also a potent activator of the vanilloid subtype 1 (TRPV1) receptors (Ghanem et al., 2016).

The pharmacological mechanisms through which paracetamol may affect reproductive development remain unclear. Experimental data suggest that in males, reduced fetal testicular production of testosterone is likely to be contributory: gestational exposure of male rats to paracetamol reduces intratesticular testosterone concentrations (Kristensen et al., 2011, Kristensen et al., 2012), and a similar effect has been reported in human fetal testicular tissue xenografted into castrate mice exposed to paracetamol at the equivalent of a human therapeutic dose for 7 days (Driesche et al., 2015). However, 1–3 days of paracetamol exposure (at 10^−5^–10^−4^ mmol/L, equivalent to clinically therapeutic serum levels) had no effect on testosterone production by human fetal testes cultured *in vitro*, instead suppressing INSL3 levels in a dose-dependent manner (Mazaud-Guittot et al., 2013). Parallel determinations of *ex vivo* testicular endocrine/paracrine activity suggest that the anti-androgenic effects of paracetamol are independent of INSL3 production, prostaglandin production, Leydig cell numbers, and gonocyte apoptosis (Kristensen et al., 2012; Albert et al., 2013). A rat study reported reduced transcription of certain steroidogenic enzyme genes (*Cyp11a1*, *Cyp17a1*) in fetal testes following exposure to high-dose paracetamol during the MPW (Driesche et al., 2015), which is consistent with steroidogenic profiling data demonstrating impaired conversion of progesterone to 17α-hydroxyprogesterone by CYP17A1, and possibly other downstream enzymes, in human adrenocortical carcinoma cells exposed to paracetamol *in vitro* (Holm et al., 2015). However, the mechanisms by which paracetamol might alter expression of such genes are unknown.

## Epidemiological Studies on Offspring Sexual Development

### Genital Anomalies

Cryptorchidism and hypospadias are the commonest urogenital anomalies in the male newborn, and are risk factors for both low semen quality and testicular germ cell cancer in later life (Hughes and Acerini, 2008). Several population-based epidemiological studies have examined associations between maternal paracetamol exposure and cryptorchidism in male offspring (Table 1).

**TABLE 1:** Reported associations between gestational paracetamol exposure and male offspring reproductive outcomes in epidemiological studies.

Study	Design	Population^a^	Paracetamol Exposure Assessment Method	Proportion of Mothers Taking Paracetamol	Male Offspring Endpoint(s)	Associations
Aselton (Aselton et al., 1985)	Prospective cohort study	United States, n = 6,509 infants (∼50% male)	Health care cooperative pharmacy records	350/6,509 (5%) during first trimester	Cryptorchidism, hypospadias (at birth)	Exposure during first trimester: no associations
Correy (Correy et al., 1991)	Prospective cohort study	Australia, n = 56,037 infants (∼50% male)	Doctor-completed obstetric/perinatal audit forms during the first trimester	1,849/56,037 (3%) during first trimester	Cryptorchidism, hypospadias (at birth)	Exposure during first trimester: no associations
Rebordosa (Rebordosa et al., 2008)	Prospective cohort study	Denmark, n = 88,142 infants (∼50% male)	Self-administered questionnaire at enrolment; 4 follow-up computer-assisted telephone interviews	26,424/88,142 (30%) during first trimester	Cryptorchidism, hypospadias (at birth)	Exposure during first trimester: no associations
Jensen (Jensen et al., 2010)	Prospective cohort study	Denmark, n = 47,400 male infants	Self-administered questionnaire; 3 computer-assisted telephone interviews	22,449/47,400 (47%) any time during pregnancy	Cryptorchidism (at birth)	Exposure during first and second trimesters, or for >4 weeks during GA 8–14 weeks: increased hazard of cryptorchidism
Kristensen (Kristensen et al., 2011)	Prospective cohort study	Denmark and Finland, n = 491 + 1,463 male infants	Self-administered questionnaire and/or computer-assisted telephone interview in third trimester	Danish mothers completing self-administered questionnaire: 127/784 (16%) any time during pregnancy. Danish mothers undergoing computer-assisted telephone interview: 233/490 (48%) any time during pregnancy, 56/398 (14%) during first trimester, 95/398 (24%) during second trimester. Finnish mothers: not stated for paracetamol alone; 619/1,463 (42%) for paracetamol and/or aspirin and/or ibuprofen any time during pregnancy	Cryptorchidism, hypospadias (at birth)	Exposure for >2 weeks (but not <2 weeks) during first and second trimesters: increased odds of cryptorchidism; no association for hypospadias
Philippat (Philippat et al., 2011)	Prospective cohort study	France, n = 903 male infants	Midwife-administered questionnaire at GA 24–28 weeks and shortly after birth	Not stated for paracetamol alone; 804/903 (89%) for paracetamol and/or aspirin and/or ibuprofen any time during pregnancy	Cryptorchidism (at birth)	No association
Wagner-Mahler (Wagner-Mahler et al., 2011)	Nested case-control study	France, n = 95 + 188 male infants	Self-administered questionnaire in early postnatal period	Not stated for paracetamol alone; 62/283 (22%) for paracetamol and/or aspirin any time during pregnancy	Cryptorchidism (at 0, 3, and 12 months of age)	No associations
Snijder (Snijder et al., 2012)	Prospective cohort study	Netherlands, n = 3,184 male infants	3 prenatal questionnaires (at GA 12, 20, and 30 weeks)	184/2,090 (9%) during GA 0–14 weeks, 465/2,864 (16%) during GA 14–22 weeks, 349/2,709 (13%) during GA 20–32 weeks	Cryptorchidism, hypospadias (at 0–48 months of age)	Exposure during GA 14–22 weeks (but not <14 or 20–32 weeks): increased odds of cryptorchidism; no association for hypospadias
Feldkamp (Feldkamp et al., 2010)	Case-control study	United States, n = 11,610 + 4,500 male infants	Computer-assisted telephone interview after birth	7,499/16,110 (47%) during first trimester	Hypospadias	Exposure during first trimester: no associations
Lind (J. N. Lind et al., 2013)	Case-control study	United States, n = 1,537 + 4,314 male infants	Computer-assisted telephone interviews 6 weeks to 24 months after EDD	3,470/5,851 (59%) from 1 month before to 4 months after conception	Hypospadias (second- or third-degree)	No association
Interrante (Interrante et al., 2017)	Case-control study	United States, n = 29,078 + 10,962	Interview at 6 weeks to 24 months after EDD	46–47% during first trimester, 45% during second trimester, 42–45% during third trimester	Hypospadias	No association
Fisher (Fisher et al., 2016)	Prospective cohort study	United Kingdom, n = 681 male infants	Self-administered questionnaire during the perinatal period	224/676 (33%) any time during pregnancy, 68/186 (37%) during GA 8–14 weeks	AGD, SPL, testicular descent (at 0, 3, 12, 18, and 24 months of age)	Exposure during GA 8–14 weeks (but not <8 or >14 weeks): shorter AGD but not SPL or testicular descent
Lind (D. V. Lind et al., 2017)	Prospective cohort study	Denmark, n = 557 male infants	Self-administered questionnaires at recruitment (GA 10–27 weeks) and at GA 28 weeks	399/1,027 (39%) during GA 0–29 weeks	AGD, penile width (at 3 months of age)	Exposure to both paracetamol and NSAIDs during pregnancy: shorter AGD but not penile width
Navarro-Lafuente (Navarro-Lafuente et al., 2021)	Prospective cohort study	Spain, n = 277 male infants	Researcher-administered questionnaire at GA 32–36 weeks	171/277 (62%) any time during pregnancy, 102/277 (37%) during first trimester, 112/277 (40%) during second trimester, 96/176 (55%) during third trimester	AGD (at birth)	No association
Ernst (Ernst et al., 2019)	Prospective cohort study	Denmark, n = 7,478 boys (and n = 7,888 girls)	Self-administered questionnaires at recruitment, 4 computer-assisted telephone interviews at GA 17 and 32 weeks and 6 months after birth	8,606/15,822 (54%) any time during pregnancy	Child-reported Tanner stages, axillary hair growth, age at voice break and first ejaculation (every 6 months from 11.5 years of age)	Exposure during pregnancy: no association in boys; earlier puberty attainment in girls

a For case-control studies, numbers of infants are stated as n = cases + controls. AGD, anogenital distance; SPL, stretched penile length; GA, gestational age; EDD, estimated date of delivery; NSAIDs, non-steroidal anti-inflammatory drugs. Only studies that investigated paracetamol as an independent exposure have been included.

The risk of cryptorchidism with maternal gestational use of paracetamol, ibuprofen and aspirin (acetylsalicylic acid) was studied in Denmark (Jensen et al., 2010). In this large prospective cohort study, 47% of the mothers were exposed some time during pregnancy to paracetamol. Exposure to paracetamol, but not ibuprofen or aspirin, in the first and second trimesters or during 8–14 weeks of gestation was associated with increased risk of cryptorchidism (hazard ratio for first and second trimesters 1.33, 95% confidence interval [CI] 1.00–1.77), although there was no clear dose-response relationship.

A smaller joint Denmark- and Finland-based study showed an association with paracetamol exposure during the second trimester and cryptorchidism, but only in the Danish cohort. The use of mild analgesics was higher in mothers whose son had cryptorchidism compared to those whose son did not (64 vs 55%, adjusted odds ratio [OR] 1.43, 95% CI 0.73–2.79) (Kristensen et al., 2011). As the prevalence of cryptorchidism in Finland was lower than in Denmark (2.4 vs 9.4%), the authors argued that the apparent lack of association in the Finnish cohort reflected a lower statistical power. Furthermore, there was no association with hypospadias in this study. Similarly, a study from the Netherlands also showed an increased prevalence of cryptorchidism in offspring exposed to paracetamol between 14 and 22 weeks of gestation (adjusted OR 1.89, 95% CI 1.01–3.51) (Snijder et al., 2012).

These findings are in contrast to the previous studies conducted in larger cohorts from the Danish National Birth Defect study (Rebordosa et al., 2008) and the Tasmania obstetric and perinatal audit (Correy et al., 1991). In addition, no association between intrauterine paracetamol exposure and cryptorchidism was found in two small studies from France (Philippat et al., 2011; Wagner-Mahler et al., 2011). Rather, cryptorchidism at birth was associated with other factors such as instrumental delivery, inguinal hernia, urogenital malformations, and paternal history of cryptorchidism. Maternal use of paracetamol during pregnancy was less common in this population (22%) (Wagner-Mahler et al., 2011) than in most other populations studied in the past 2 decades (30–62%) (Rebordosa et al., 2008; Feldkamp et al., 2010; Jensen et al., 2010; Kristensen et al., 2011; J. N. Lind et al., 2013; Fisher et al., 2016; Interrante et al., 2017; D. V. Lind et al., 2017; Ernst et al., 2019; Navarro-Lafuente et al., 2021), with the exception of one Dutch study (9–16%) (Snijder et al., 2012).

No positive association with maternal paracetamol use and hypospadias has been reported in epidemiological studies to date (J. N. Lind et al., 2013; Feldkamp et al., 2010; Interrante et al., 2017), although non-steroidal anti-inflammatory drugs (NSAIDs) have been reported to be a risk factor for hypospadias as well as other birth defects such as gastroschisis, cleft lip and palate, neural tube defects, and congenital heart disease (Interrante et al., 2017).

### AGD

Some studies of prenatal paracetamol exposure have utilised AGD as a more sensitive tool to evaluate effects on androgen activity during the MPW. Unlike cryptorchidism or hypospadias, AGD is not usually assessed clinically, but its properties as an easily measured, continuous variable could potentially detect differences associated with paracetamol exposure with smaller sample sizes than those required for rare, categorical outcomes (Kristensen et al., 2011). Use of AGD measurements in a modestly-sized UK cohort, the Cambridge Baby Growth Study (CBGS) (Thankamony et al., 2009), indicated that exposure to paracetamol during 8–14 weeks of gestation, but not <8 weeks or >14 weeks of gestation, was associated with shorter AGD in male infants from birth to 24 months of age, suggesting that fetal masculinisation was impaired. Penile length and cryptorchidism were not significantly associated with paracetamol intake in this study, although there was a trend to reduced penile length in males exposed during 8–14 weeks of gestation (Fisher et al., 2016). A similar trend was shown in a Danish study, but observed only with combined maternal use of paracetamol and NSAIDs; the relevant paracetamol exposure had occurred later in gestation (the second trimester) (D. V. Lind et al., 2017). However, a small more recent Spanish report was not able to replicate these findings (Navarro-Lafuente et al., 2021).

### Puberty

Of great importance is knowing whether such exposure to analgesics *in utero* affects later development at puberty and adult life. This was addressed by a Danish study which assessed pubertal outcomes in both males and females using self-administered questionnaires completed approximately every 6 months (Ernst et al., 2019). Paracetamol-exposed females attained puberty slightly earlier than non-exposed females and importantly, the effect was dose-dependent. In contrast, no association with pubertal development was found in males. The mechanism of early puberty attainment in girls is unclear. Pubertal timing is multifactorial, influenced by genetic factors, fat mass, and exposure to the endocrine disruptors, particularly oestrogenic compounds and antiandrogens. The effect of endocrine disruptors on GnRH secretion, and therefore initiation of puberty, is controversial and still inconclusive (Abreu and Kaiser 2016).

Plans are currently in place to conduct a similar analysis of participants in CBGS who are now of pubertal age and beyond. While this non-invasive self-assessment approach is a relatively blunt instrument to assess puberty, the results will be important for mothers concerned about having taken medication during their pregnancy.

## Experimental Studies

A number of experimental studies have aimed to further explore the impact of fetal exposure to paracetamol on offspring gonadal and genital development using animal, *in vitro*, and *ex vitro* (xenograft) models (Bauer et al., 2021). A detailed discussion of these studies is beyond the scope of this review, however.

There have also been a number of animal studies investigating the effects of aniline (phenylamine), a precursor of paracetamol, on fetal gonadal development. Aniline is an industrial chemical widely used in the production of rubber, pesticides, and colorants used in food, cosmetics, and textiles. Aniline is rapidly converted to paracetamol by the liver (Modick et al., 2016) and paracetamol has been detected in the urine of people who have not taken paracetamol and who are not industrial workers, suggesting ubiquitous exposure to environmental sources (Modick et al., 2014). In rodent studies, fetal exposure to aniline has been reported to exert similar effects to paracetamol: a reduction in AGD in pups and altered male sexual behaviour in adulthood (Holm et al., 2015; Hay-Schmidt et al., 2017). While exposure to paracetamol in humans is predominantly from pharmaceutical products, it is implicit to also consider and further study the role of chemicals such as aniline to which humans are exposed.

## Discussion

The possible adverse effects of taking paracetamol in early pregnancy on fetal reproductive development have long been debated. Experimental studies using animals and human tissue have provided increasing evidence to suggest that paracetamol is capable of altering gonadal and genital development (Bauer et al., 2021). However, confirmation of these findings from epidemiological studies have been inconsistent. Possible reasons include differences between experimental animals (typically rodents) and humans (physiology and dose equivalence, for example), and the limitations of an *in vitro* experiment to replicate the *in vivo* environment of local paracetamol concentrations operating in relation to factors such as endocrine and paracrine signalling and homeostatic mechanisms.

Epidemiological studies are complicated by many other factors. Gestational paracetamol exposure in all studies to date has been measured indirectly, usually by using questionnaires (that are often unvalidated) to collect data retrospectively. This approach risks introducing inaccurate recollection, and non-differential exposure misclassification (Feldman et al., 1989). Furthermore, there may be confusion with use of other analgesic drugs. This is particularly the case when questioning post-dates when the outcome of interest has been assessed, for example cryptorchidism or hypospadias. There are many compounding factors to consider: inaccurate diagnoses such as location of testis descent leading to outcome misclassification; and the problem of selection bias. The latter is illustrated by participants in CBGS who were significantly less likely to be smokers compared to the UK population, and therefore may have been exposed to fewer pollution-related and industrial endocrine disruptors (Fisher et al., 2016). Other factors to consider are demographic and genetic differences between geographically separated populations and conditions that underlie the indications to paracetamol use. The environment contains thousands of chemicals to which pregnant women and the developing fetus are exposed. They include substances, usually not measured in the environment, with endocrine-disrupting properties such as anti-androgenic compounds, for example dichlorodiphenyldichloroethylene (DDE), phthalates, and NSAIDs (Torres-Sanchez et al., 2008; Swan et al., 2015; D. V.; Lind et al., 2017). While the effect from each compound may individually be minimal at low concentrations, there is evidence of a ‘cocktail’ effect through exposure in combination, especially over a prolonged period during a vulnerable stage of development (Gore et al., 2015).

In conclusion, it is evident that robust epidemiological research is required to assess the danger to human health from compounds with endocrine-disrupting properties such as paracetamol (Ho et al., 2022). In addition to extrapolating from experimental studies such as xenograft models, large prospective surveys of paracetamol usage are required through pregnancy (especially clinical indication, dosage, duration and gestational interval) with follow-up of offspring to puberty and adulthood. Pregnant women are bombarded with warnings about exposure to a range of potential risks to their baby, ranging from caffeine intake, various foods including artificial sweeteners, hair dyes and cosmetics, to drinking water. So what should the pregnant woman do if she has a headache or a fever? The evidence for clinically significant reproductive effects in humans exposed to paracetamol is limited and inconclusive. However, since there is some evidence for a dose-dependent toxic effect of paracetamol, the precautionary principle is the sensible approach to adopt: using the lowest dose of paracetamol for the minimum period to relieve their discomfort during periods of ill health.
